# Comprehensive UPLC-MS/MS Profiling of Bioactive Phenolics and Their MYB Regulatory Networks in Wild and Cultivated Strawberries

**DOI:** 10.3390/molecules31091517

**Published:** 2026-05-03

**Authors:** Muhammad Junaid Rao, Kangjian Song, Sijiu He, Shirong He, Yuanqiao Li, Ima Mulyama Zainuddin, Yubo Chen, Xinnian Du, Wei Liu, Munsif Ali Shad, Maryam Tahira, Xiande Duan, Bingsong Zheng, Liuyuan Bao, Shunqiang Yang, Mingzheng Duan

**Affiliations:** 1 State Key Laboratory for Development and Utilization of Forest Food Resources, Zhejiang A&F University, Hangzhou 311300, China; mjr@zafu.edu.cn; 2 Advanced Institute of Ecological Agriculture and Biodiversity on the Yunnan-Guizhou Plateau/Yunnan Key Laboratory of Smart Villages and Agri-Cultural-Tourism Integration, Zhaotong University, Zhaotong 657000, China; 202315120109@stu.ztu.edu.cn (K.S.); 202415120117@stu.ztu.edu.cn (S.H.); duxinnian108@163.com (X.D.); yl2024030029@ztu.edu.cn (X.D.); 47015@ztu.edu.cn (L.B.);; 3 Research Centre for Genetic Engineering, National Research and Innovation Agency (BRIN), Cibinong 16912, Indonesia; imam024@brin.go.id; 4 Institute of Pomology, Jilin Academy of Agricultural Sciences, Gongzhuling 136100, China; 5 National Key Laboratory for Germplasm Innovation & Utilization of Horticultural Crops, College of Horticulture and Forestry, Huazhong Agricultural University, Wuhan 430070, China

**Keywords:** strawberry, phenolic acids, UPLC-MS/MS, MYB transcription factors, metabolomics, antioxidant activity, natural products, functional foods

## Abstract

Phenolic compounds are vital bioactive constituents in fruits, yet modern strawberry breeding has often reduced their diversity. Here, we employed a multi-omics approach integrating UPLC-MS/MS-based metabolomics and RNA-seq transcriptomics to investigate the divergence in phenolic profiles and their transcriptional regulation between a wild strawberry (*Fragaria nilgerrensis*, HM) and three cultivated varieties (white ‘Danxue’ (DX), pink ‘Fenyu’ (FY), and red ‘Red Face 99’ (RF)). The wild HM genotype exhibited higher antioxidant activity and a significantly more complex phenolic profile, dominated by high-abundance galloylated and benzoylated glucosides (e.g., digallic acid methyl ester, salicylic acid-2-*O*-glucoside) that were largely absent or depleted in cultivated fruits. In contrast, the cultivated varieties displayed specialized yet simplified profiles: DX accumulated hydroxycinnamoyl galactonic acids, FY was enriched in feruloylated glucosides, and RF was characterized by coumaroyl-glucose derivatives. Transcriptomic analysis identified a set of MYB transcription factors (e.g., *FxaYL_531g0581170*, *FxaYL_642g0175720*) significantly upregulated in wild HM, with strong correlations to key bioactive phenolics such as 4-hydroxybenzoate and salicylic acid derivatives. These findings illustrate how selective breeding has reshaped phenolic composition through alterations in MYB regulatory networks. The wild strawberry germplasm thus represents a valuable natural reservoir for biofortification strategies aimed at restoring the nutritional and functional quality of modern strawberry cultivars.

## 1. Introduction

Strawberry (*Fragaria × ananassa*), a globally valued fruit, is praised not only for its vibrant color and distinct flavor but also for its considerable nutritional value [1]. This value is largely attributed to a rich range of bioactive compounds, including phenolic acids, flavonoids, anthocyanins, and vitamin C, which collectively contribute to its high antioxidant capacity [2]. Epidemiological and clinical studies have linked the consumption of strawberries and their phenolic constituents to a variety of health benefits, ranging from anti-inflammatory and anti-cancer effects to improved cardiovascular and neurological health [2,3]. The sensory appeal and health-promoting properties of strawberries are intrinsically linked to their phytochemical composition, which is influenced by genetic background, cultivation practices, environmental conditions, and post-harvest handling. However, the modern commercial strawberry breeding has primarily been driven by consumer and market demands for superior agronomic traits. Decades of intensive selection have focused on enhancing fruit size, yield, visual appeal, shelf-life, texture (hardness), and sweetness, often at the unintended expense of nutritional quality and phytochemical diversity [4]. This narrowing of genetic diversity through modern breeding has created a significant metabolic gap between commercial cultivars and their wild relatives.

Wild *Fragaria* species, having evolved under diverse environmental pressures, often possess a more complex and abundant array of secondary metabolites, which confer enhanced stress tolerance and defensive capabilities [5]. Consequently, many valuable bioactive compounds prevalent in wild genotypes have been diminished or lost in modern cultivated varieties, raising critical questions about the metabolic opportunity cost of modern strawberry breeding [6]. Among these wild genetic resources, *Fragaria nilgerrensis*, known locally in China as ‘Huangmao’ (HM) or “yellow-haired” strawberry, represents a particularly promising yet underexplored reservoir of genetic diversity [5,7]. Unlike the familiar red cultivated strawberry, the wild *F. nilgerrensis* produces fruits known for their resilience and distinct metabolic profile.

Phenolic acids are potent bioactive compounds ubiquitously found in fruits, where they contribute significantly to sensory attributes like color and astringency while also conferring robust antioxidant, antimicrobial, and anti-inflammatory benefits [8,9,10,11,12]. In fruits like blueberries and apples, hydroxycinnamic acids and benzoic acid derivatives are well-documented for enhancing shelf-life by preventing oxidative rancidity and for promoting human health through free radical scavenging [13,14,15]. Berries like blackberries and raspberries are packed with beneficial compounds like ellagic and gallic acid [16,17]. Cranberries stand out as a particularly rich source of natural benzoic acid [18]. Similarly, blueberries and strawberries are excellent sources of health-promoting phenolic acids, including hydroxycinnamic acids such as caffeic and p-coumaric acids [19,20,21,22]. While phenolic acids are well-studied in many berries, their variation across wild and cultivated strawberries (white, pink, and red) remains poorly understood. This research gap leaves the effect of modern breeding on phenolic composition unclear. This research identifies health-promoting phenolic acids that were lost during modern breeding and are preserved in wild relatives, providing a foundational roadmap for breeding nutritionally enhanced strawberries.

MYB transcription factors (TFs) are among the largest gene families in plants and are widely recognized for their regulatory roles in phenolic biosynthesis and plant stress responses [23,24]. The phenylpropanoid pathway, in particular, gives rise to a broad spectrum of secondary metabolites, including phenolic acids, flavonoids, proanthocyanidins, and lignins [25,26,27]. Phylogenetic and functional studies have revealed that MYB activators associated with this pathway can be grouped into distinct subfamilies, each responsible for modulating the expression of downstream biosynthetic genes and thereby influencing the accumulation of phenolic acids and flavonoids [28]. Among these, the R2R3-MYB subfamily is the most abundant and functionally diverse in higher plants [29,30]. In *Arabidopsis thaliana*, three well-characterized R2R3-MYB genes (*AtMYB113*, *AtPAP1*, and *AtPAP2*) have been shown to activate phenolic biosynthetic genes, leading to enhanced pigment accumulation during leaf development [23,31]. Similarly, in the cultivated strawberry (*Fragaria × ananassa* Duch.), anthocyanin biosynthesis is predominantly regulated by *FaMYB* genes [24,26,32]. For instance, *FaMYB5* has been identified as a positive regulator of pigmented phenolic compounds accumulation in both fruit skin and flesh [33]. In wild strawberry (*Fragaria vesca*), FvMYB10 and FvMYB41 are strongly associated with anthocyanin accumulation, while interactions between FvMYB and FvbHLH proteins promote proanthocyanidin biosynthesis [34]. These findings underscore the central role of MYB transcription factors as master regulators of phenolic metabolism and stress resilience [35]. While MYB regulators of pigments and flavonoids are well-characterized in strawberries, the MYB genes governing phenolic acid biosynthesis in both wild and cultivated genotypes remain largely unexplored. This knowledge gap significantly limits our understanding of the regulatory mechanisms shaping phenolic acid diversity across strawberry varieties.

In this study, we employ a targeted comparative approach, integrating advanced UPLC-MS/MS metabolomics with RNA-seq transcriptomics to: (1) comprehensively quantify and compare the profiles of bioactive phenolic acids and overall antioxidant capacity in one wild (*F. nilgerrensis*, HM) and three cultivated strawberry varieties (White ‘Danxue’, Pink ‘Fenyu’, and Red ‘Red Face 99’); and (2) identify and correlate the expression patterns of MYB transcription factors with the accumulation of specific phenolic metabolites. By understanding the metabolic and regulatory differences between wild and cultivated strawberries, this study provides valuable information on bioactive phytochemicals and highlights their regulatory genes for developing nutritionally enhanced, consumer-acceptable strawberries, effectively bridging functional genomics with food technology to advance bio-fortification in the fruit industry.

## 2. Results

### 2.1. Varietal Differences in Bioactive Compounds and Antioxidant Activity

**Figure 1:**
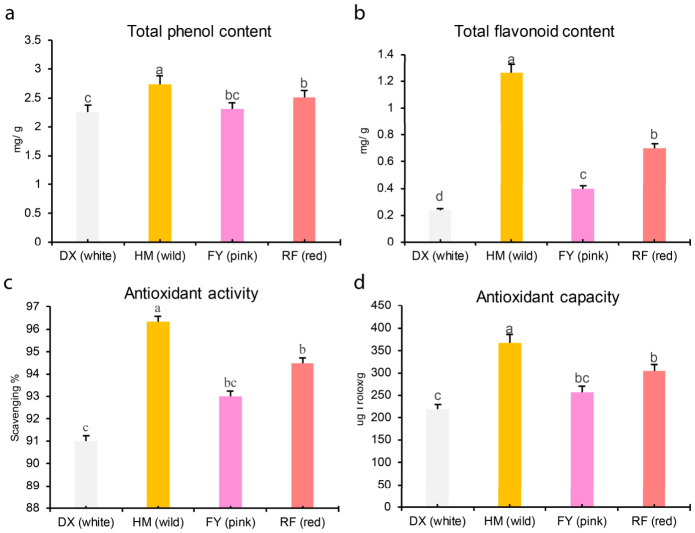
Comparative analysis of bioactive content and antioxidant activity in four strawberry genotypes. Bar graphs display (**a**) total phenolic content (mg GAE/g FW), (**b**) total flavonoid content (mg CE/g FW), (**c**) DPPH free radical scavenging rate (%), and (**d**) antioxidant capacity (µg Trolox/g FW) for wild HM and cultivated varieties DX (white), FY (pink), and RF (red). Values represent mean ± SD (*n* = 3 biological replicates). Different lowercase letters indicate significant differences (Fisher’s LSD post hoc test, *p* < 0.05).

The wild strawberry (HM) exhibited higher phenolic contents and antioxidant properties, achieving the highest values in total phenolic content (2.74 mg/g), total flavonoid content (1.26 mg/g), DPPH radical scavenging rate (96.33%), and antioxidant capacity (367.09 µg Trolox/g) (Figure 1a–d). Among the cultivated varieties, the red-fruited ‘Red Face 99’ (RF) ranked highest across all measured parameters, followed by the pink ‘Fenyu’ (FY) and the white ‘Danxue’ (DX), which consistently showed the lowest values (Figure 1a–d).

**Figure 2:**
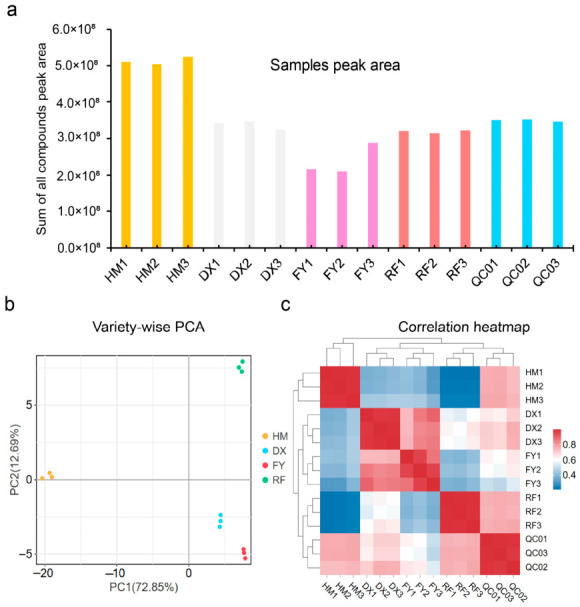
Phenolic acid metabolic profiles of four strawberry genotypes. (**a**) Total peak area of phenolic compounds per sample (*n* = 3 biological replicates). (**b**) Principal component analysis (PCA) score plot of genotype groups: HM (wild), DX (white), FY (pink), and RF (red). (**c**) Pearson correlation heatmap of phenolic acid profiles among biological replicates and quality control (QC) samples. Red indicates positive correlation; blue indicates negative correlation.

The sum of all phenolic compound peak areas revealed significant quantitative differences among the strawberry varieties. The wild genotype *Fragaria nilgerrensis* (HM) demonstrated the highest total phenolic abundance, markedly exceeding the three cultivated varieties. Among the cultivars, DX and RF contained a higher phenolic content than FY (Figure 2a). The low variance of the quality control (QC) samples confirms the high reproducibility and reliability of the analytical methodology. Principal Component Analysis (PCA) of the metabolite profiles revealed clear separation among the four strawberry varieties, with the first two principal components (PC1 and PC2) accounting for 85.54% of the total variance (PC1: 72.85%, PC2: 12.69%). The HM clustered distinctly from the three cultivated varieties (DX, RF, FY), indicating an obvious divergence in their metabolic composition (Figure 2b). Among the cultivated varieties, DX (white), FY (pink), and RF (red) also form separate clusters, demonstrating significant intra-species biochemical diversity (Figure 2b). The correlation heatmap showed strong reproducibility within each variety’s replicates and a clear metabolic divergence between wild (HM) and cultivated (DX, FY, RF) genotypes (Figure 2c). The tight clustering of QC samples validated the analytical reliability of the phenolic acid data (Figure 2c).

### 2.2. Wild and Cultivated Strawberries Exhibit Distinct Phenolic Acid Profiles

**Figure 3:**
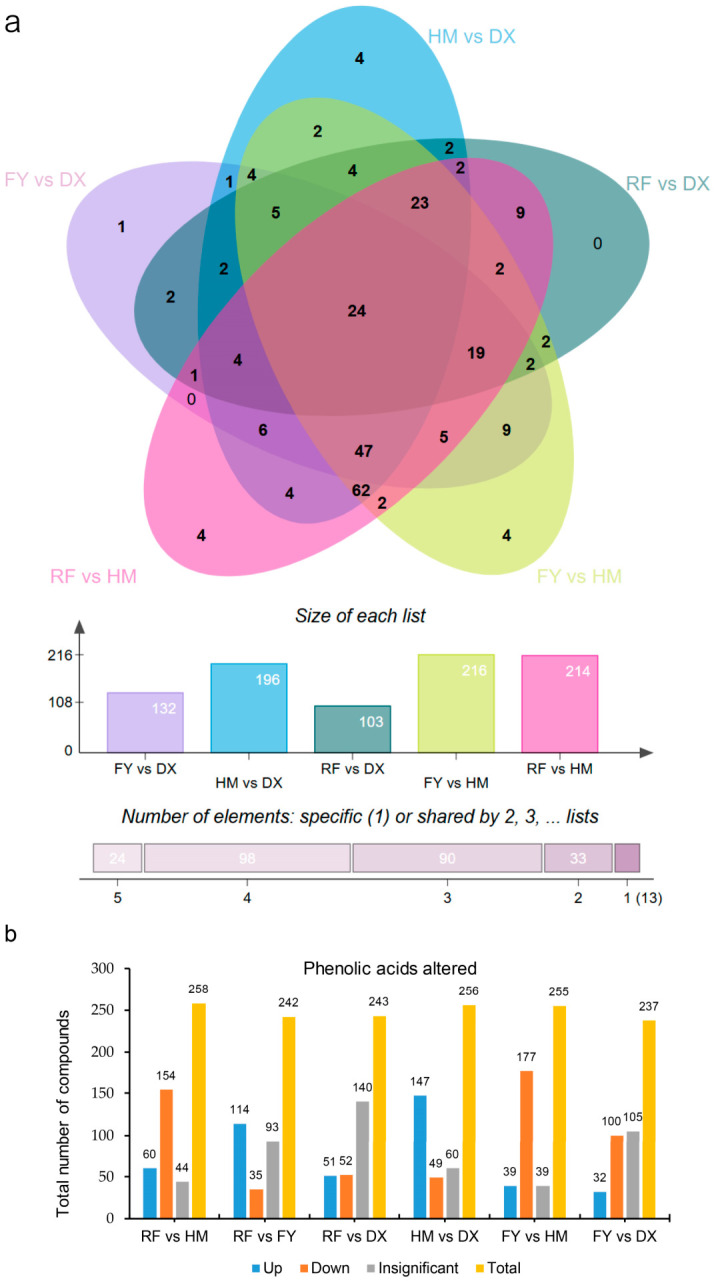
Differential accumulation of phenolic acids among strawberry varieties. (**a**) Venn diagram showing numbers of phenolic acids significantly altered across five pairwise comparisons. (**b**) Numbers of upregulated (blue), downregulated (red), and unaltered (gray) phenolic acids in each comparison. Significance was determined by VIP score > 1.0, |log_2_ fold change| ≥ 1, and FDR-adjusted *p* < 0.05.

A comparative metabolomic analysis revealed variably shared phenolic acid alterations across five pairwise comparisons of strawberry varieties. The Venn diagram illustrates that a core set of 24 phenolic acids was commonly found across all comparisons (Figure 3a). Moreover, the comparisons involving the wild genotype HM (e.g., FY vs. HM, RF vs. HM, and HM vs. DX) contained the largest number of uniquely altered compounds (216, 214, and 196, respectively), highlighting their distinct phenolic profile dominated by the loss of these compounds in cultivated varieties. Conversely, comparisons between cultivated varieties (e.g., FY vs. DX and RF vs. DX) showed a distinct alteration of phenolic acids (132 and 103, respectively), indicating conserved phenolic metabolism post-domestication (Figure 3a). The comparison-specific analysis of different strawberry groups revealed that the wild germplasm HM served as a distinct phenolic reservoir, with the number of compounds down-regulated in cultivated varieties when compared to it (e.g., 154 down in RF vs. HM, 177 down in FY vs. HM). Conversely, the comparison between the two cultivated varieties, RF and FY, showed a bias towards up-regulation (114 up vs. 35 down), indicating a significant divergence in their phenolic profiles (Figure 3b). The comparisons involving DX often resulted in a high number of insignificant changes (e.g., 140 in RF vs. DX, 105 in FY vs. DX), suggesting a more conserved phenolic metabolism between this white-fruited and the red and pink (Figure 3b). These findings highlight that modern breeding and selection for agronomic traits have significantly reshaped the phenolic acid composition, primarily through the loss of diverse compounds from the wild germplasm.

### 2.3. Clustering and Grouping of Phenolic Acids in Strawberry Fruits

**Figure 4:**
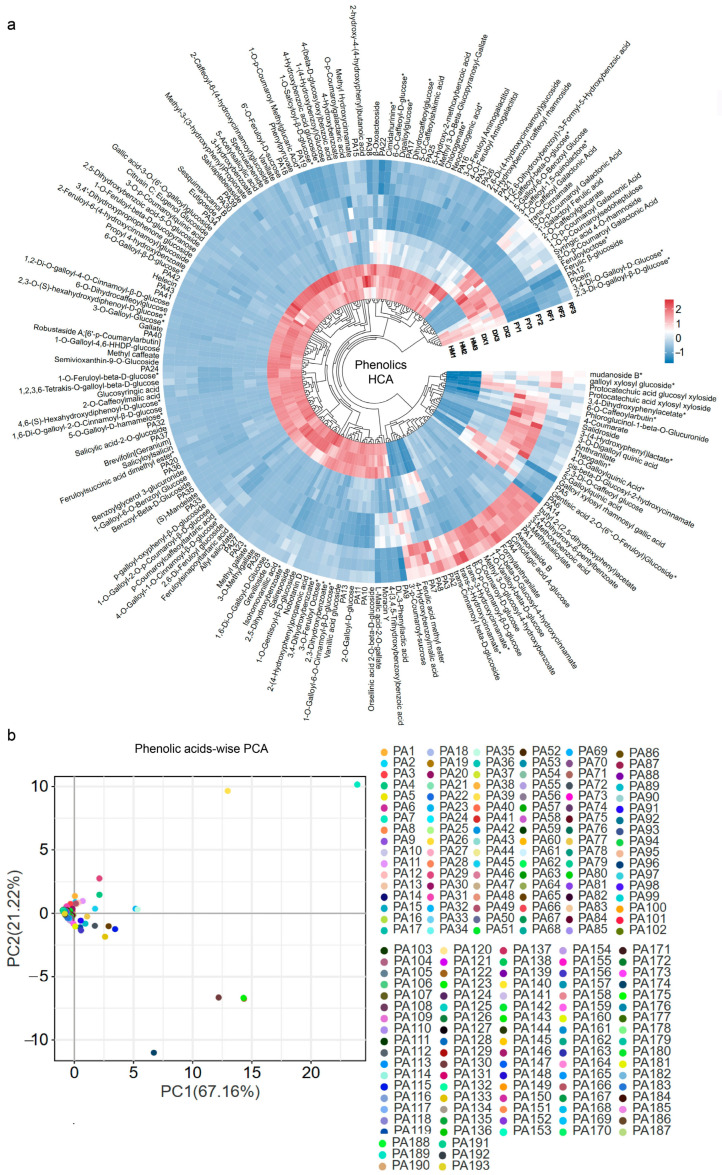
Clustering and distribution of phenolic acid profiles across four strawberry varieties. (**a**) Hierarchical cluster analysis heatmap; rows represent normalized phenolic acid abundances, columns represent samples. Compound identifiers (PA1–PA43) correspond to Supplementary Table S3. Scale colors represent the up and down regulation of compunds from red to blue, respectively. (**b**) Compound-wise PCA score plot of phenolic acids; identifiers PA1–PA193 correspond to Supplementary Table S4.

Hierarchical cluster analysis (HCA) of the 194 phenolic compounds revealed an obvious divergence in the phytochemical profiles between the wild and cultivated strawberry varieties. The wild HM strawberry variety exhibited a significantly more complex and abundant phenolic signature, with nearly 140 compounds (including complex, high-molecular-weight phenolic esters and benzoic acid derivatives) present in higher concentrations compared to all three cultivated types (DX-white, FY-pink, and red-RF) (Figure 4a). Among the cultivated strawberries, a color-associated trend was observed. The red strawberry (RF) contained the highest levels of 25 specific phenolic acids, positioning it as the most phenol-rich cultivated genotype. The higher phenolic content in RF indicates that breeding for color (anthocyanin accumulation) may be loosely linked to the preservation of some branches of the phenylpropanoid pathway. The pink (FY) and white (DX) varieties showed comparatively reduced phenolic density, with higher abundance of 14 and 19 compounds, respectively (Figure 4a).

Based on the PCA results, the phenolic acids demonstrate a clear separation and distribution pattern across the two principal components (Figure 4b). The two principal components (PC1 and PC2) account for 88.38% of the total variance (PC1: 67.16%, PC2: 21.22%). The majority of the compounds are clustered closely together in the x-axis and y-axis intersection point, except for 14 compounds. The PCA results, which correlate with the HCA results, suggest that the HM wild strawberry is a valuable genetic resource for breeding programs aimed at enhancing the bioactive compounds and nutritional value of commercial strawberries.

### 2.4. Percentage Abundance of Key Bioactive Phenolic Acids in Strawberry Fruits

**Figure 5:**
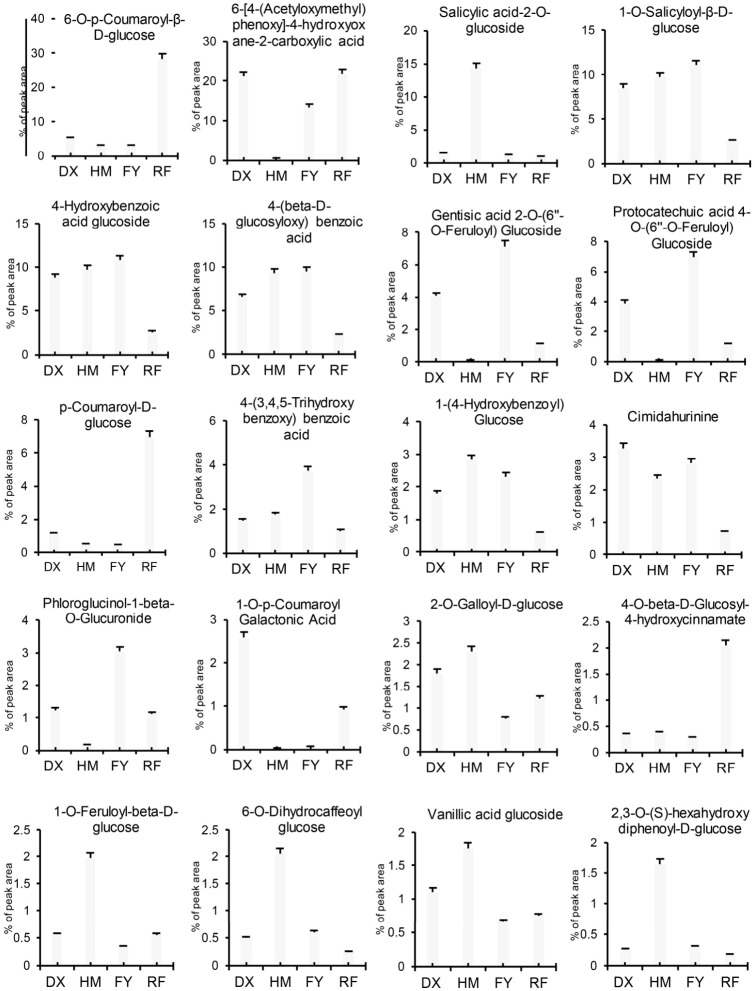
Relative abundance (% of total peak area) of selected phenolic acids and their derivatives in white (DX), wild (HM), pink (FY), and red (RF) strawberries. Values represent means ± standard deviation based on triplicate analyses (*n* = 3).

The relative abundance of key phenolic acids and their derivatives (% peak area) revealed distinct metabolic profiles among the four strawberry varieties. The cultivated variety DX was notably enriched in specific hydroxycinnamic acid conjugates, with high abundances of 1-O-p-Coumaroyl Galactonic Acid (2.58%) and 6-[4-(Acetyloxymethyl)phenoxy]-4-hydroxyoxane-2-carboxylic acid (21.22%), compounds associated with antioxidant activity (Figure 5). In contrast, the wild germplasm HM accumulated higher levels of glucosides, such as salicylic acid-2-*O*-glucoside (14.34%), a compound linked to plant defense and potential anti-inflammatory properties, highlighting its value as a genetic reservoir for bioactive phenolics. The FY showed a higher accumulation of feruloylated glucosides, such as protocatechuic acid 4-O-(6″-O-feruloyl)glucoside (6.96%) and gentisic acid 2-O-(6″-O-feruloyl)glucoside (7.11%), which may contribute to its sensory properties. The red RF was characterized by an exceptionally high accumulation of 6-O-p-Coumaroyl-β-D-glucose (28.39%) and p-Coumaroyl-D-glucose (6.96%) (Figure 5).

### 2.5. Wild and Cultivated Genotypes Exhibit Divergent and Specialized Phenolic Profiles

The wild HM genotype showed significantly elevated levels of complex, high-molecular-weight phenolic compounds, including diverse galloylated and benzoylated glucosides such as digallic acid methyl ester, gentisic acid 5-O-β-D-(6′-O-galloyl)-glucopyranoside, methyl gallate, methyl hydroxycinnamate, and 1-Galloyl-6-O-Benzoyl Glucose, which were significantly lower in cultivated varieties (Table 1). In contrast, DX accumulated notably high levels of hydroxycinnamoyl galactonic acid derivatives such as 1-O-p-coumaroyl galactonic acid and 1-O-caffeoyl galactonic acid, which were nearly absent in FY. FY exhibited higher levels of compounds such as butyl 2-(2,5-dihydroxyphenyl)acetate and rosmarinate, while RF showed increased accumulation of the same phenylacetate derivative alongside other unique compounds such as methyl 2-O-(4-hydroxybenzoyl)-2,4,6-trihydroxyphenylacetate (Table 1). These results showed that cultivated varieties exhibit specialized metabolite profiles that may enhance specific flavor or post-harvest properties, while the wild germplasm retains structurally complex phenolics associated with potent antioxidant activity.

### 2.6. MYB Expression Profiling and Correlation with Phenolic Acids

**Figure 6:**
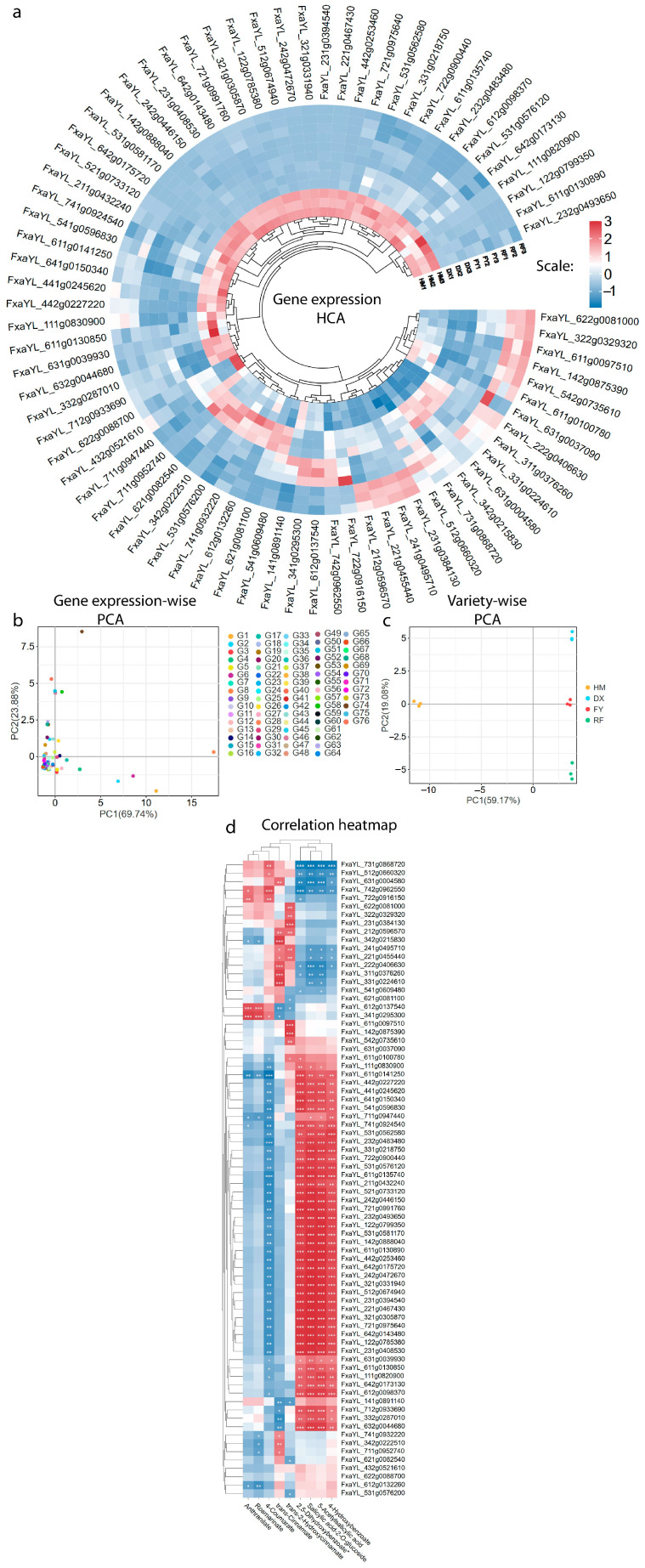
MYB transcription factor expression patterns reveal variety-specific regulatory profiles. (**a**) Hierarchical clustering heatmap of MYB gene expression. (**b**) Gene-wise PCA of MYB expression. (**c**) Sample-wise PCA illustrating transcriptional divergence between HM and cultivated genotypes. (**d**) Pearson correlation matrix between MYB expression and key phenolic acid abundances. Significance levels: * *p* < 0.05, ** *p* < 0.01, *** *p* < 0.001. Red, positive correlation; blue, negative correlation; white, non-significant (|r| < 0.5).

Hierarchical cluster analysis of MYB transcription factor gene expression revealed distinct varietal clustering patterns. The wild strawberry (HM) formed a unique cluster characterized by the significant upregulation of 43 MYB genes, with many (*FxaYL_231g0394540*, *FxaYL_221g0467430*, *FxaYL_442g0253460*, *FxaYL_721g0975640*, *FxaYL_531g0581170*, *FxaYL_142g0888040*, *FxaYL_642g0175720*, etc.) showing expression levels higher than in the cultivated varieties (Figure 6a). Among the cultivated genotypes, the red strawberry (RF) and white strawberry (DX) often clustered together, generally exhibiting higher transcript levels for a different set of MYBs compared to the pink (FY) variety, which frequently showed the lowest expression among the cultivated types (Figure 6a). This transcriptomic divergence strongly suggests that the rich phenolic profile of the wild HM variety is regulated by a specialized MYB regulatory network governing these valuable health-promoting compounds.

Principal Component Analysis (PCA) of MYB transcription factor expression profiles effectively segregated both the genes and the four strawberry varieties into distinct clusters, confirming a strong genotype-specific regulatory pattern (Figure 6b,c). The wild variety (HM) formed a tightly grouped cluster that was completely separate from the three cultivated varieties (DX, FY, RF), indicating a fundamental divergence in its transcriptional regulatory network, which aligns with the hierarchical clustering analysis (Figure 6a–c). Among the cultivated genotypes, the RF and DX varieties were far apart, whereas the FY variety occupied a unique intermediate position (Figure 6c). The high cumulative variance explained by the first two principal components (69.74% for PC1 and 23.88% for PC2 in the gene-wise analysis; 59.17% and 19.08%, respectively, in the variety-wise analysis) reveals that the expression patterns of these MYB genes are the key factor of transcriptional variation, effectively unique the genetic backgrounds and their associated phenolic phenotypes.

Our correlation analysis revealed a network of MYB transcription factor genes with strong, statistically significant positive relationships to the accumulation of key phenolic acids in strawberries (Figure 6d). Several genes associated with specific phenolic acids: for instance, *FxaYL_742g0962550* showed a highly significant correlation (*** *p* < 0.001) with 4-coumarate (also known as p-coumaric acid, which is high in FY, Figure 4a), while the genes *FxaYL_612g0137540* and *FxaYL_341g0295300* were both strongly linked (*** *p* < 0.001) to the levels of anthranilate and rosmarinate (Figure 6d). Furthermore, the gene *FxaYL_342g0222510* emerged as a key regulator significantly correlated with trans-cinnamate (also called trans-cinnamic acid, high in DX, Figure 4a). Beyond these specific relationships, the genes *FxaYL_531g0581170*, *FxaYL_142g0888040*, and *FxaYL_642g0175720* showed high significance in their correlation (*** *p* < 0.001) with multiple metabolites, including 4-hydroxybenzoate, salicylic acid-2-*O*-glucoside, 5-acetylsalicylic acid, and 2,5-dihydroxybenzoate (Figure 6d). This correlation is particularly consistent with the metabolic profile of the HM variety, in which these beneficial phenolic compounds are abundant (Figure 4a).

**Figure 7:**
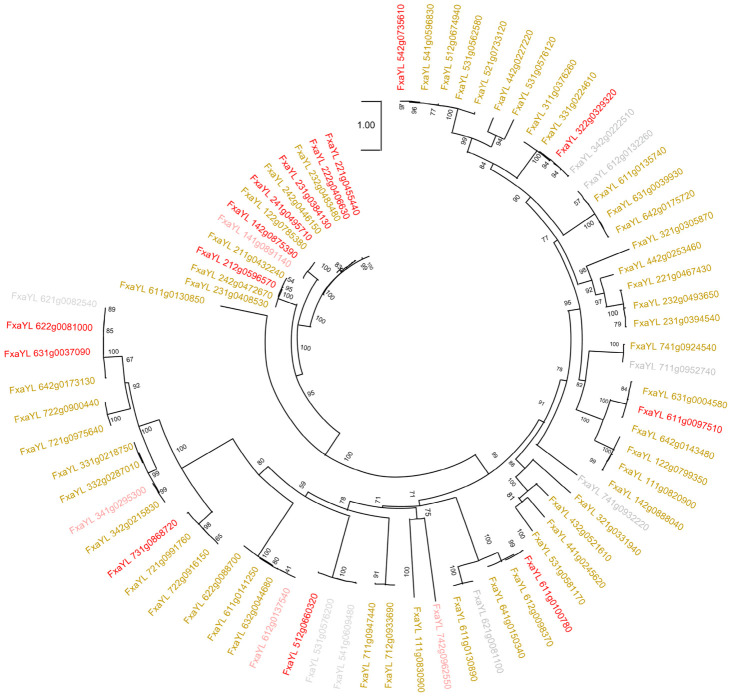
Phylogenetic clustering of DEGs encoded MYB proteins. Yellow, red, pink, and light gray colored protein labels, respectively, represent DEGs associated with HM, RF, FY, and DX genotypes.

In order to contextualize *MYB* expressional divergence across strawberry genotypes, we performed phylogenetic classification of MYB proteins (Figure 7). Our analysis exhibited, with a few exceptions of proteins FxaYL_221g0455440/222g0406630, 622g0081000/631g003709, and 342g0222510/612g0132260, the distribution of MYB proteins was random at large into different phylogenetic groups.

**Figure 8:**
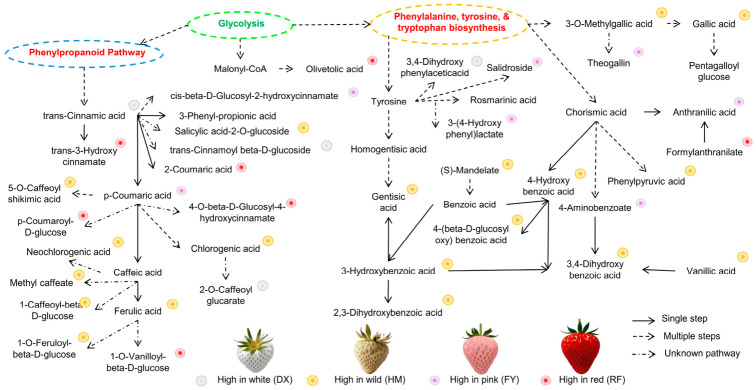
Schematic of the phenolic acids biosynthetic pathway highlighting metabolic variations between wild HM and cultivated strawberries (DX, FY, and RF). Compounds enriched in each genotype are indicated by colored circles.

The phenolic acid biosynthesis pathway reveals that the HM strawberry exhibits a distinct and chemically complex profile, rich in phenolic acid derivatives (e.g., benzoic acid derivatives ‘4-hydroxybenzoate’, gallic acid derivatives ‘digalloyl esters and galloyl-glucoses’, and hydroxycinnamic acid esters such as coumaroyl ‘methyl hydroxycinnamate’, and feruloyl glycosides (Figure 8), which are strongly associated with potent antioxidant and plant defense properties. In contrast, the common cultivated variety FY is a rich source of diverse hydroxycinnamic acid derivatives, such as 1-O-p-coumaroyl galactonic acid, 1-O-caffeoyl galactonic acid, and DX, high in rosmarinate and anthranilate (Figure 6), which are valued for their functional food and health-related properties. Overall, the pathway showed that the wild strawberry exhibited a distinct phytochemical signature characterized by a high abundance of phenolic acid compounds with increased antioxidant capacity (Figure 1 and Figure 8).

## 3. Discussion

Our study provides compelling evidence that selective breeding has significantly reshaped the phenolic acid profiles and underlying regulatory networks in strawberry fruits. Our integrated metabolomic and transcriptomic analyses reveal that the wild HM genotype contains a higher and chemically complex phenolic portfolio compared to cultivated varieties, characterized by an abundance of high-molecular-weight galloylated and benzoylated glucosides (Figure 4a, Figure 5 and Figure 7, Table 1). These findings align with the growing body of literature that suggests modern breeding programs, while successful in enhancing agronomic traits such as yield and fruit size [36], have inadvertently led to a reduction in phytochemical diversity and antioxidant capacity (Figure 1). For instance, previous studies have documented a decline in phenolic compounds in cultivated strawberries relative to wild relatives, often attributed to genetic bottlenecks and selection for consumer-preferred traits such as sweetness and appearance [6,24]. Our results extend these observations by systematically quantifying the specific phenolic acids lost in cultivated varieties vs. wild and linking these metabolic shifts to divergent expression patterns of MYB transcription factors. We acknowledge that this study examined only one wild accession of *Fragaria nilgerrensis* (HM); therefore, the observed metabolic profile may not fully represent the intraspecific diversity of this species or other wild *Fragaria* relatives. Future work incorporating multiple wild accessions from varied geographic origins will be needed to validate the generalizability of these findings.

The distinct clustering of HM in both PCA and HCA analyses underscores its unique metabolic identity, rich in compounds such as salicylic acid-2-*O*-glucoside, methyl gallate, and various feruloylated and coumaroylated derivatives (Figure 4a,b). These compounds are not only potent antioxidants but also play roles in plant defense mechanisms [37,38], suggesting that the wild genotype may possess enhanced stress resilience. This is consistent with reports that wild *Fragaria* species often exhibit greater tolerance to abiotic and biotic stresses, partly mediated by elevated levels of defense-related secondary metabolites [39]. The cultivated varieties, by contrast, display specialized but simplified profiles. For example, the red cultivar (RF) accumulated high levels of coumaroyl-glucosides, while the DX (white) and FY (pink) varieties were enriched in hydroxycinnamoyl galactonic acids and phenylacetate derivatives, respectively. This specialization may reflect breeding efforts aimed at optimizing specific sensory attributes or post-harvest characteristics, albeit at the expense of metabolic diversity.

The significant upregulation of MYB genes in HM, including candidates such as *FxaYL_231g0394540* and *FxaYL_531g0581170*, strongly implies that these transcription factors may regulate the phenylpropanoid pathway in wild strawberries; however, functional validation is required to confirm these results. The strong positive correlations between specific MYBs and metabolites such as p-coumaric acid, anthranilate, and salicylic acid derivatives provide mechanistic clues about how phenolic diversity is associated with transcription factor expression patterns. Furthermore, the phylogenetic classification of MYB was not consistent with the expression patterns across the understudied genotypes (Figure 7). We speculate that, instead of lineage-specific coding sequence, cis-regulatory variations in the promoters and epigenetic modifications might drive genotype-specific expression of MYB genes. Previous studies have identified MYB transcription factors (MYB TF) as critical regulators of flavonoid and anthocyanin biosynthesis in strawberries [32,33]; however, our work expands this role to hydroxycinnamic and benzoic acid derivatives, highlighting a broader regulatory influence of MYB TF (Figure 6). The transcriptional divergence between wild and cultivated genotypes suggests that selection for agronomic traits has altered the expression or function of these regulatory genes, thereby narrowing metabolic output.

From a food science and evolutionary perspective, these findings have significant implications. The higher antioxidant activity and chemically complex phenolic profile of the wild HM genotype position it as a valuable genetic resource for biofortification strategies aimed at enhancing the health-promoting properties of strawberries [40,41,42]. Incorporating HM traits into breeding programs could help restore nutritional quality without compromising key agronomic traits [43,44]. Wild *Fragaria* species often exhibit greater tolerance to abiotic and biotic stresses, partly mediated by elevated levels of defense-related secondary metabolites. This is exemplified by *F. nilgerrensis*, which has been shown to possess excellent drought resistance linked to robust antioxidant defense mechanisms [45,46,47,48]. Therefore, the observed metabolic richness in HM likely reflects an evolutionary adaptation to environmental pressures, and the reintegration of this genetic diversity could revitalize both the nutritional profile and stress resilience of cultivated strawberries [47,49]. Furthermore, the MYB regulatory network identified here presents concrete molecular targets; selection of beneficial MYB alleles or the use of gene-editing techniques to modulate their expression could accelerate the development of nutrient-dense strawberry varieties [50,51,52]. By bridging metabolomics and transcriptomics, our work provides a foundational roadmap for developing next-generation strawberry varieties with optimized health benefits, aligning with consumer demand for natural, functional foods and supporting more sustainable agricultural practices.

## 4. Materials and Methods

### 4.1. Plant Material Collection, Cultivation Environment, and Sample Processing

Four strawberry genotypes were utilized in this study: three commercial *Fragaria × ananassa* cultivars designated as RF (‘Red Face 99’), FY (‘Fenyu’), and DX (‘Danxue’), obtained from Dongji Luyuan Agriculture and Animal Husbandry Co., Ltd. in Dandong, China, along with wild *Fragaria nilgerrensis* samples collected from Yongshan County, Yunnan Province during October 2023. The genotypes were vegetatively propagated through stolon division and cultivated in controlled greenhouse facilities at Zhaotong University.

Standardized seedlings were established in August 2023 using individual containers measuring 19 × 15 × 72 cm, with five plants per container to maintain optimal plant density. Three container replicates were prepared for each genotype. The growing medium comprised Pindstrup peat moss (10–30 mm size granules; Denmark) supplemented with 20% vermiculite and decomposed sheep manure compost to optimize substrate composition and nutrient availability. Manual cross-pollination was performed using fine brushes to ensure uniform fruit set. Mature fruits were collected in March 2024 when physiological ripeness was achieved across all genotypes. Physiological ripeness was determined based on genotype-specific criteria: for the red cultivar RF, full red coloration of the fruit surface; for the pink cultivar FY, uniform pink coloration; for the white cultivar DX, fully developed white fruit; and for the wild HM genotype, complete transition from green to pale white with soft texture. Additionally, fruits were harvested at approximately 30–35 days post-anthesis to ensure comparable developmental stages across genotypes.

Representative fruits were selected from the most vigorous container of each variety to reduce developmental variation. Berries from all five plants within the selected container were combined, carefully cleaned with deionized water to remove external debris, air-dried on sterilized filter paper, and sectioned into uniform fragments (approximately 0.5 cm^3^) using sterile surgical blades. Samples were immediately immersed in liquid nitrogen for rapid freezing, then pulverized to fine powder using mechanical grinding. Three independent biological replicate containers were processed in this manner for each genotype (*n* = 3). Each biological replicate consisted of pooled fruit tissue from five plants grown in a single container. The homogenized material from each biological replicate was aliquoted into sterile cryogenic vials and maintained at −80 °C before transcriptomic, biochemical, and metabolomic analyses.

### 4.2. Chemical Reagents

The chemical reagents used across the assays included: Folin–Ciocalteu reagent (Sigma-Aldrich, St. Louis, MO, USA), DPPH (2,2-diphenyl-1-picrylhydrazyl) (Aladdin, Shanghai, China), NaNO_2_, Al(NO_3_)_3_, and NaOH (Macklin, Shanghai, China) for flavonoid analysis, DNS (3,5-dinitrosalicylic acid) (Yuanye Bio-Technology, Shanghai, China), ninhydrin, and sulfosalicylic acid (Solarbio, Beijing, China), HPLC grade acetonitrile, methanol, and ethanol (Fisher Scientific, Waltham, MA, USA), UPLC-MS grade formic acid (Merck, Darmstadt, Germany), standard Trolox (Sigma-Aldrich, USA), UPLC-MS internal standard ‘2-chlorophenylalanine’ (Purity: 98%, CAS: 14091-11-3; Manufacturer: Bailingwei, Beijing, China, LBCOR15), and gallic acid (TCI Chemicals, Tokyo, Japan). Phenolic content quantification was achieved by G0118W kit sourced from Suzhou Geruisi Biotechnology Co., Ltd., (Suzhou, China; www.geruisi-bio.com). All solvents and standards were of analytical or HPLC grade.

### 4.3. Assessment of Total Phenolic Content in Strawberry Fruit Samples

Three independent biological replicates were analyzed for each genotype (*n* = 3 biological replicates per variety). Each biological replicate consisted of pooled fruit tissue from five plants grown in a single container. The total phenolic content of strawberry fruits was determined using a commercial Total Phenols Assay Kit (Folin–Ciocalteu method; Catalog No. G0117W; Suzhou Geruisi Biotechnology Co., Ltd., Suzhou, China; www.geruisi-bio.com) following the manufacturer’s instructions. Briefly, approximately 0.1 g of frozen fresh fruit tissue was homogenized in 1.5 mL of 60% ethanol. The homogenate was incubated at 60 °C for 2 h with shaking, then centrifuged at 12,000 rpm for 10 min at 25 °C. The supernatant (10 µL) was mixed with 50 µL of Reagent 1 (Folin–Ciocalteu reagent) and incubated in the dark for 3 min, followed by the addition of 50 µL of Reagent 2 (sodium carbonate solution). Distilled water (90 µL) was added to bring the final volume to 200 µL. After 30 min incubation at room temperature in the dark, absorbance was measured at 760 nm using a microplate reader. A standard curve was prepared using the gallic acid standard provided in the kit. Total phenolic content was expressed as mg of gallic acid equivalent per gram of fresh weight (mg GAE/g FW).

### 4.4. Assessment of Total Flavonoid Content in Strawberry Fruit Samples

Total flavonoid content (TFC) in strawberry fruits (*n* = 3 biological replicates per variety) was determined employing a colorimetric assay based on the NaNO_2_-Al(NO_3_)_3_-NaOH method, utilizing a commercial TFC assay kit (G0118W, Geruisi-Bio, China; available at: https://www.geruisi-bio.com/product/G0118W?goodsno=G0118W; Accessed on 22 May 2024). Fresh fruit samples (0.1 g) were mechanically homogenized in 1.5 mL of 60% ethanol solution. The homogenate was extracted at 60 °C for 2 h under continuous agitation, followed by centrifugation at 12,000× *g* for 10 min. From the resulting supernatant, 50 μL was transferred to a reaction mixture containing 15 μL of reagent 1 (5 g/100 mL *w*/*v* NaNO_2_ solution). Following a 6 min incubation period at 25 °C, 30 μL of reagent 2 (10 g/100mL *w*/*v* Al(NO_3_)_3_ solution) was added, immediately followed by 105 μL of reagent 3 (4 g/100mL *w*/*v* NaOH solution). After a 15 min reaction period at room temperature, spectrophotometric measurements were conducted at 510 nm wavelength using a SpectraMax iD5 microplate reader (Molecular Devices, San Jose, CA, USA).

Quantification was achieved by comparison with a standard calibration curve prepared using rutin equivalent concentrations ranging from 0 to 1 mg/mL. The TFC was calculated according to Equation (1):

TFC (mg/100 g fresh weight) = [0.6 × (Asample − Acontrol + 0.0049)/(W × V × D)] × 100(1)

where W represents the sample fresh weight (g), V denotes the extraction solvent volume (1.5 mL), and D indicates the dilution factor (no dilution applied in this study).

### 4.5. Antioxidant Activity and Capacity in Strawberry Fruit Samples

The antioxidant capacity of the strawberry fruits (*n* = 3 biological replicates per variety) was evaluated by measuring their ability to scavenge the stable 2,2-diphenyl-1-picrylhydrazyl (DPPH) free radical. The assay was performed using a commercial DPPH Radical Scavenging Ability Assay Kit (G0128W, Grace Biotechnology Co., Ltd., Suzhou, China). First, a DPPH working solution was prepared by dissolving the provided reagent powder in 12.5 mL of absolute ethanol, as instructed in the kit manual. This solution was stored in the dark at 4 °C until use. An 80% (*v*/*v*) methanol solution was also prepared as the extraction solvent. For sample preparation, 0.1 g of frozen fruit tissue from each sample was accurately weighed. The tissue was then homogenized with 1 mL of the pre-chilled 80% methanol solution. The extraction was carried out using an ultrasonic bath set at 60 °C and 200–300 W for 30 min, with manual vortex mixing performed at 5 min intervals to ensure efficient extraction. After sonication, the volume of any lost solvent was replenished with 80% methanol to maintain a 1 mL volume. The extracts were then centrifuged at 12,000 rpm for 10 min at room temperature to pellet insoluble material, and the resulting supernatant was carefully collected for the assay.

For each sample, three reaction systems were prepared: a Test tube, a Sample Control tube, and a Blank Control tube. In the Test tube, 150 µL of the sample supernatant was mixed with 150 µL of the DPPH working solution. The Sample Control tube contained 150 µL of the sample supernatant mixed with 150 µL of 80% methanol (to account for the sample’s inherent color). The Blank Control tube contained 150 µL of 80% methanol mixed with 150 µL of the DPPH working solution (to represent the initial absorbance of the DPPH radical). All tubes were thoroughly vortexed after reagent addition and then incubated at 25 °C in the dark for 30 min to allow the reaction to proceed to completion. Following incubation, the tubes were centrifuged at 12,000 rpm for 5 min. Finally, 200 µL of the supernatant from each tube was transferred to a 96-well microplate. The absorbance of each well was measured at 517 nm using a microplate reader. The DPPH radical scavenging activity was calculated as a percentage using the formula:

Scavenging Activity (%) = [1 − (A_sample − A_sample_control)/A_blank] × 100%

where A_sample is the absorbance of the Test tube, A_sample_control is the absorbance of the Sample Control tube, and A_blank is the absorbance of the Blank Control tube.

To express the results in standard Trolox Equivalent (TE) units, a calibration curve was constructed. A Trolox stock solution (1 mg/mL) was prepared in methanol and diluted to 0, 5, 10, 15, 20, and 25 µg/mL. These standard solutions underwent the exact same assay procedure as the samples. The antioxidant capacity of the strawberry samples was then quantified using this curve and the formula:

DPPH Radical Scavenging Capacity (µg Trolox/g FW) = 0.351 × (Scavenging% − 0.7084)/W

where W is the fresh weight in grams. All extractions and measurements were performed in triplicate (*n* = 3).

### 4.6. Comprehensive Phenolic Acid Profiling of Strawberry Fruit Samples

#### 4.6.1. Sample Processing and Metabolite Isolation

Fresh strawberry samples (*n* = 3 biological replicates per variety) underwent freeze-drying treatment utilizing vacuum lyophilization equipment (Scientz-100F, Ningbo Scientz Biotechnology Co., Ltd., Ningbo, China) for a duration of 63 h. The dehydrated materials were then homogenized into fine powder using a high-frequency ball mill (MM 400, Retsch, Haan, Germany) set at 30 Hz for 90 s. Approximately 30 mg of the powdered sample was accurately weighed on a precision balance (MS105DΜ, Mettler-Toledo International Inc., Zurich, Switzerland) and placed into designated extraction vessels. The metabolomic study of strawberry fruits was conducted in collaboration with Wuhan Metware Biotechnology Co., Ltd. (Wuhan, China; available at: http://www.metware.cn/) employing ultra-performance liquid chromatography coupled with tandem mass spectrometry (UPLC-MS/MS) methodology [53].

The metabolite isolation process involved adding 1500 μL of pre-cooled (−20 °C) methanol solution (70% aqueous) supplemented with internal standard compounds (2-chlorophenylalanine at 1 ppm concentration) to each sample, achieving a sample-to-solvent ratio of 1:50 (*w*/*v*). The samples underwent periodic vortex agitation (30 s intervals every 30 min) for 6 cycles to ensure optimal extraction efficiency. Subsequently, the samples were centrifuged (Eppendorf Model 5424R, Eppendorf AG, Hamburg, Germany) at 12,000× *g* for 3 min at 4 °C, and the supernatant was carefully collected. The extracts were filtered through 0.22 μm membrane filters and transferred to injection vials for UPLC-MS/MS analysis.

#### 4.6.2. Chromatographic Separation Conditions

Chromatographic separation was performed using an ExionLC™ AD UPLC system (SCIEX, Framingham, MA, USA) coupled to a triple quadrupole mass spectrometer. An Agilent SB-C18 reversed-phase column (1.8 μm, 2.1 mm × 100 mm) was used and maintained at 40 °C. The mobile phase consisted of (A) 0.1% formic acid in water and (B) 0.1% formic acid in acetonitrile. The gradient elution program was as follows: 0–9.0 min, 5–95% B; 9.0–10.0 min, 95% B; 10.0–11.1 min, 95–5% B; and 11.1–14.0 min, 5% B for column re-equilibration. The flow rate was 0.35 mL/min, and the injection volume was 2 μL. The column eluate was directed to an electrospray ionization (ESI) source operating in both positive and negative ionization modes.

#### 4.6.3. Mass Spectrometric Analysis Parameters

Mass spectrometric detection was performed using an ESI-QTRAP-MS system with optimized operational settings: source temperature maintained at 500 °C; electrospray voltages configured at +5500 V for positive ion mode and −4500 V for negative ion mode; gas pressures for nebulizer (GS1), auxiliary heater (GS2), and curtain gas were set to 50, 60, and 25 psi, respectively; collision gas pressure was maintained at high level. Multiple reaction monitoring (MRM) analyses were conducted in a triple quadrupole configuration utilizing nitrogen as a collision gas under medium-pressure conditions. Individual optimization of declustering potential (DP) and collision energy (CE) parameters was performed for each MRM transition to achieve maximum analytical sensitivity and specificity. Scheduled MRM acquisition was implemented, whereby specific transition monitoring occurred within predetermined retention time intervals corresponding to target metabolite elution windows, following established protocols [53]. Metabolite identification was accomplished through comparison of precise mass transitions, parent ion (Q1) and fragment ion (Q3) masses, and fragmentation patterns against reference databases (comprehensive metabolite data provided in Supplementary Table S1). Mass spectrometric detection was carried out using a SCIEX QTRAP^®^ 6500+ (SCIEX, Framingham, MA, USA) triple quadrupole mass spectrometer equipped with an ESI source. The ESI parameters were optimized as follows: ion source temperature, 500 °C; ion spray voltage, +5500 V (positive mode) and −4500 V (negative mode); curtain gas, 25 psi; nebulizer gas (GS1) and heater gas (GS2), 50 and 60 psi, respectively; collision gas, medium; collision-activated dissociation (CAD) parameter, high. Data acquisition was performed in multiple reaction monitoring (MRM) mode. For each target metabolite, the optimal precursor ion (Q1), product ion (Q3), declustering potential (DP), and collision energy (CE) were determined using authentic standards and compiled into an in-house MRM library, following established protocols [53]. MRM transitions were scheduled into specific retention time windows to maximize data points per peak and ensure optimal sensitivity.

#### 4.6.4. Data Processing, Quality Control, and Metabolite Identification

Raw data files were processed using Analyst^®^ 1.6.3 and MultiQuant™ 3.0.2 software (SCIEX) for peak integration, calibration, and quantification. Metabolite identification was based on matching retention time and MRM transitions with the in-house MWDB (MetWare Database) library (www.metware.cn). Following the guidelines of the Metabolomics Standards Initiative (MSI), identification confidence was assigned at three levels: Level 1 (identified metabolites), confirmed by matching retention time and MS/MS spectrum with authentic standards; Level 2 (putatively annotated compounds), based on spectral matching with public or commercial databases without confirmation by authentic standards; and Level 3 (putatively characterized compound classes), based on characteristic MS/MS fragmentation patterns. In this study, the majority of phenolic acid identifications corresponded to Level 2 confidence, with a subset validated at Level 1 using authentic standards. The complete list of identified compounds, including MSI confidence levels, is provided in Supplementary Table S1.

For relative quantification, the peak area of each metabolite was normalized against the peak area of the internal standard (2-chlorophenylalanine) in the same sample. Quality control (QC) samples, prepared by pooling equal aliquots from all experimental samples, were injected at regular intervals (every 5–10 samples) throughout the analytical sequence. The stability of the analytical system was assessed by evaluating the coefficient of variation (CV) of metabolites detected in the QC replicates; data were considered acceptable when over 85% of metabolites exhibited a CV < 0.5.

### 4.7. RNA Extraction, Sequencing, and Analysis

Total RNA was extracted from strawberry fruit samples (*n* = 3 biological replicates per variety) using the TRIzol reagent kit (Invitrogen, Carlsbad, CA, USA). Library preparation was carried out using the NEBNext Ultra™ RNA Library Prep Kit (NEB, Ipswich, MA, USA) following standard manufacturer instructions. The sequencing was performed on the Illumina HiSeq 2500 platform (Illumina, Shanghai, China) to generate paired-end reads, with subsequent removal of low-quality sequences during filtering. Berry Hekang Biotech. (Beijing, China) handled the RNA assembly and library construction processes.

For transcriptome analysis, we selected the Fragaria vesca v6 genome as our reference sequence (retrieved 4 December 2023, from http://eplantftp.njau.edu.cn/Fragaria/F._vesca/F._vesca_v6.0/; accessed on 12 January 2026), paired with the corresponding genome annotation file (Fragariavescav6genome.gff). We verified gene ID compatibility between our raw transcriptomic data and the reference genome. The RNA-seq raw datasets have been submitted to the China National GeneBank (CNGB; https://db.cngb.org/cnsa; accessed on 16 July 2024) under project accession CNP0007631. We mapped clean reads to the reference genome using the HISAT2 software.

The gene annotation strategy incorporated several comprehensive databases: clusters of orthologous groups of proteins (KOG/COG), gene ontology (GO), protein family (Pfam), NCBI non-redundant protein sequences (Nr), NCBI non-redundant nucleotide sequences (Nt), Kyoto Encyclopedia of Genes and Genomes Ortholog (KO), and Swiss-Prot. RNA-seq data underwent quality control procedures, including adapter trimming with cutadapt and quality evaluation using fastqc [54]. The differentially expressed genes (DEGs) were identified using cuffdiff, applying a threshold of |Log2FoldChange| ≥ 1 and *p*-value < 0.05. GO enrichment analysis of DEGs was performed using the GOseq R package (version 2.18.0) with the Wallenius noncentral hypergeometric distribution [55]. We also conducted KEGG pathway enrichment analysis using KOBAS 2.0 software to examine biological pathways influenced by the DEGs. Normalized expression values (FPKM) for all MYB genes analyzed in this study are provided in Supplementary Table S5.

### 4.8. Multivariate Statistical Methods

All experiments were performed using three independent biological replicates per genotype (*n* = 3), with each replicate comprising pooled fruit from five plants. Technical replicates were averaged prior to analysis; only biological replicates were treated as independent units. Biochemical data (Figure 1) were analyzed with Statistix 8.1, followed by Fisher’s LSD post hoc test (*p* < 0.05). Data is presented as mean ± SD.

For metabolomics (Figure 2, Figure 3, Figure 4 and Figure 5, Table 1), Z-score normalized data were subjected to PCA and HCA using OmicShare tools (accessed on 1 March 2025; http://www.omicshare.com/tools) [56]. Differentially accumulated metabolites were identified by OPLS-DA (VIP > 1.0) [57] and two-tailed unpaired Student’s *t*-test with Benjamini–Hochberg FDR correction (*p* < 0.05) (Supplementary Table S2) [58], combined with a fold-change threshold (|log_2_FC| ≥ 1) [59]. MetaboAnalystR v1.0.0 in R v4.2.1 was used for OPLS-DA [60]. Venn diagrams were generated with EVenn (accessed in 3 April 2025; https://www.bic.ac.cn/EVenn/#/) [56].

RNA-seq DEGs (Figure 6) were identified using Cuffdiff v2.2.1 (|log_2_FC| ≥ 1, FDR < 0.05; Benjamini–Hochberg correction), as detailed in Section 4.7. Pearson correlations between metabolites and MYB expression (Figure 6d) were computed using OmicShare [56]; significance was assessed via two-tailed *t*-test (* *p* < 0.05, ** *p* < 0.01, *** *p* < 0.001). Non-significant correlations (*r* < 0.5) are shown in white.

### 4.9. Phylogenetic Analysis

The DEGs MYB genes encoded protein sequences were extracted from the *Fragaria vesca* v6 genome. The MYB protein sequences were used for phylogenetic tree construction in the Linux environment. The protein sequences were aligned using MAFFT version 7. The aligned sequences were subsequently processed with ModelFinder, UFBoot2, and IQ-TREE to accurately select models, approximate bootstrap support, and generate maximum-likelihood phylogenetic trees [61,62,63,64]. MEGA12 was used for phylogenetic tree visualizations and figure generation [65].

## 5. Conclusions

This study demonstrates that modern breeding has significantly altered the phenolic acid composition and regulatory architecture in strawberries. The wild strawberry (*Fragaria nilgerrensis*) contains a chemically complex profile rich in galloylated and benzoylated glucosides, accompanied by enhanced antioxidant activity, whereas cultivated varieties displayed simplified, color-specific phenolic signatures. Transcriptomic analysis revealed MYB transcription factors were strongly upregulated in the wild genotype, with key genes (e.g., *FxaYL_531g0581170*, *FxaYL_642g0175720*) correlating positively with beneficial phenolic metabolites. These findings indicate wild strawberry germplasm as a valuable resource for reintroducing bioactive diversity into modern breeding programs. Although putative MYB regulators are identified by our association analysis, their significant roles in phenolic acid production must be confirmed through functional validation using qRT-PCR, gene silencing, overexpression, gene editing, and MYB promoter binding (Yeast 1 Hybrid, EMSA) investigations. Leveraging these genetic insights can guide the development of nutritionally enhanced strawberries with improved stability and bioactivity, offering significant potential for application in functional foods, natural preservatives, and health-focused product innovation.

## Figures and Tables

**Table 1:** Differential accumulation of characteristic phenolic acids in wild (*Fragaria nilgerrensis*) vs. cultivated (white, pink, red) strawberry fruits.

Serial No.	Compounds	DX vs. HM				
		VIP	*p*-Value	Fold Change	Log2FC	Type
1	1-O-p-Coumaroyl Galactonic Acid	1.12	0.00	39.88	5.32	up
2	1′-Galactoyl Ferulic acid	1.09	0.06	38.81	5.28	up
3	1-O-Caffeoyl Galactonic Acid	1.10	0.02	38.79	5.28	up
4	5-O-p-Coumaroyl Galactonic Acid	1.11	0.01	36.60	5.19	up
5	trans-Cinnamoyl beta-D-glucoside	1.12	0.00	36.32	5.18	up
6	Methyl gallate*	1.11	0.04	0.03	−5.04	down
7	Methyl Hydroxycinnamate	1.11	0.04	0.03	−5.08	down
8	2,3-Butanediol 2-O-(6″-O-p-coumaroyl)glucoside	1.12	0.01	0.02	−5.33	down
9	Grevilloside G*	1.12	0.02	0.02	−5.41	down
10	3-O-Feruloyl Octose*	1.12	0.00	0.02	−5.46	down
11	Robustaside A;[6′-p-Coumarylarbutin]	1.11	0.00	0.02	−5.51	down
12	3,4′-Dihydroxypropiophenone glucoside	1.10	0.02	0.02	−5.60	down
13	3-(4-hydroxyphenyl)-3-oxopropyl beta-D-glucopyranoside	1.11	0.00	0.02	−5.87	down
14	Sesquimarocanol B	1.11	0.00	0.02	−6.01	down
15	Digallic acid methyl ester	1.09	0.11	0.01	−6.22	down
16	gentisic acid 5-O-β-D-(6′-O-galloyl)-gluco-pyranoside	1.12	0.00	0.01	−6.36	down
17	2,6-Di-Feruloyl glucoside	1.12	0.01	0.01	−6.58	down
18	1-Galloyl-6-O-Benzoyl Glucose	1.12	0.00	0.00	−7.83	down
		**FY vs. DX**				
		**VIP**	***p*** **-value**	**Fold Change**	**Log2FC**	**Type**
19	butyl 2-(2,5-dihydroxyphenyl)acetate	1.24	0.00	139.45	7.12	up
20	Anthranilate	1.23	0.00	69.62	6.12	up
21	3-O-Digalloyl quinic acid	1.24	0.03	48.44	5.60	up
22	Rosmarinate	1.24	0.02	33.24	5.05	up
23	5-O-p-Coumaroyl Galactonic Acid	1.23	0.01	0.02	−5.89	down
24	1,4,6-Tri-O-galloyl-β-D-glucose	1.23	0.05	0.02	−5.99	down
25	1-O-p-Coumaroyl Galactonic Acid	1.24	0.00	0.01	−6.15	down
26	1-O-Caffeoyl Galactonic Acid	1.24	0.02	0.00	−8.42	down
27	1′-Galactoyl Ferulic acid	1.23	0.06	0.00	−8.42	down
		**RF vs. HM**				
		**VIP**	***p*** **-value**	**Fold Change**	**Log2FC**	**Type**
28	butyl 2-(2,5-dihydroxyphenyl)acetate	1.12	0.00	342.71	8.42	up
29	Methyl 2-O-(4-hydroxybenzoyl)-2,4,6-trihydroxyphenylacetate	1.12	0.00	203.06	7.67	up
30	Awsoniaside B	1.12	0.00	173.25	7.44	up
31	trans-Cinnamoyl beta-D-glucoside	1.11	0.00	34.83	5.12	up
32	5-Galloyl-6-O-Benzoyl Glucose	1.12	0.01	0.01	−7.21	down
33	3-(4-hydroxyphenyl)-3-oxopropyl beta-D-glucopyranoside	1.12	0.00	0.01	−7.57	down
34	Protocatechuic acid 4-O-(6″-O-Galloy)Glucoside	1.12	0.00	0.01	−7.64	down
35	1-Galloyl-6-O-Benzoyl Glucose	1.12	0.00	0.00	−7.79	down
36	3,4′-Dihydroxypropiophenone glucoside	1.12	0.02	0.00	−9.76	down

## Data Availability

The original contributions presented in this study are included in the article and the Supplementary Materials. The raw RNA-seq datasets generated during this study are openly available in the China National GeneBank (CNGB) Nucleotide Sequence Archive (CNSA) under project accession number CNP0007631 (available at: https://db.cngb.org/cnsa/; accessed on 12 January 2026).

## Supplementary Materials

The following supporting information can be downloaded at: https://www.mdpi.com/article/10.3390/molecules31091517/s1. Table S1: Complete UPLC-MS/MS dataset of identified phenolic acid compounds across all strawberry varieties. Table S2: Statistical analysis of phenolic acid metabolites, including fold-change, *p*-values, and VIP scores for all pairwise comparisons. Table S3: Hierarchical cluster analysis (HCA)–Compound identifiers and assigned nomenclature for phenolic acid metabolites. Table S4: Principal component analysis (PCA)–Compound identifiers and assigned nomenclature for phenolic acid metabolites. Table S5: Expression values (FPKM) of MYB transcription factor genes in four strawberry genotypes (from three independent biological replicates per genotype).
